# Increasing Use of Video Telehealth Among Veterans Experiencing Homelessness with Substance Use Disorder: Design of A Peer-Led Intervention

**DOI:** 10.1007/s41347-022-00290-2

**Published:** 2022-12-21

**Authors:** Lynn A. Garvin, Mary Alexis Greenan, E. Jennifer Edelman, Cindie Slightam, D. Keith McInnes, Donna M. Zulman

**Affiliations:** 1 Center for Healthcare Organization and Implementation Research, VA Boston Healthcare System, Boston, MA USA; 2 Department of Health Law, Policy and Management, Boston University School of Public Health, Boston, MA USA; 3 Program in Addiction Medicine, Yale School of Medicine, New Haven, CT USA; 4 Department of Internal Medicine, Yale School of Medicine, New Haven, CT USA; 5 Center for Innovation to Implementation, VA Palo Alto Health Care System, Menlo Park, CA USA; 6 Center for Healthcare Organization and Implementation Research, VA Bedford Healthcare System, Bedford, MA USA; 7 Division of Primary Care and Population Health, Stanford University School of Medicine, Stanford, CA USA

**Keywords:** Homelessness, Substance use disorder, Mental health, Video telehealth, Intervention

## Abstract

Telehealth offers promising opportunities, but also challenges, for veterans experiencing homelessness — during the COVID-19 pandemic and beyond. Recent research found low utilization of clinical video visits among homeless veterans receiving a VA tablet, and having a substance use disorder (SUD) further reduced visit likelihood. Hence, this study sought to identify unique barriers to telehealth use among veterans experiencing homelessness with a SUD and design an intervention to promote adoption. This qualitative study was guided by the Unified Theory of Acceptance and Use of Technology (UTAUT) model. The study’s three phases included veteran interviews (*N* = 28) to identify barriers and facilitators to video telehealth use and propose intervention candidates, a provider expert panel to obtain feedback on interventions, and a focus group with veterans to complete the intervention. Finally, a prototype was designed using the intervention mapping approach. Veteran interviews revealed that barriers to video telehealth included complex physical and mental health issues, lack of digital literacy, and insufficient technical support. Together, veterans and experts proposed five intervention candidates. In the end, a veteran focus group combined two candidates, peer-led digital training and motivational interviewing. Intervention mapping was used to design a “stepped care” intervention that trains and activates veterans at all skill levels. This study demonstrates how inclusion of expert and veteran views led to development of a novel intervention to support and sustain video telehealth use among veterans experiencing homeless with SUD.

## Introduction

The Veterans Affairs (VA) Healthcare System faces unique challenges in caring for the over 37,000 veterans experiencing homelessness (Henry et al., 2020; Ray et al., 2017) and an additional 1.4 million veterans at risk of homelessness (National Coalition for Homeless Veterans - Background & Statistics, 2021). Homeless veterans are a VA priority population who face elevated rates of mental illness (Ding et al., 2018), suicide (Holliday et al., 2021), substance use disorder (SUD) (Finlay et al., 2021) and have twice the rate of fatal overdose as the general population. Homeless veterans are less likely than other veterans to receive healthcare and treatment (Rosenthal et al., 2021). The COVID-19 pandemic limited social interaction and access to care (Marini et al., 2020; Tsai & Wilson, 2020), likely contributing to the increases in US drug-related deaths in 2020 and 2021 (Centers for Disease Control and Prevention, 2022).

Homelessness compounded by SUD increases life chaos and health risks (e.g., infectious disease through needle sharing) often with fatal consequences (Cox et al., 2017). Hence, it is imperative to connect veterans experiencing homelessness with SUD to healthcare, including primary, specialty, and SUD treatment options. Fortunately, a number of virtual behavioral health treatments (Connolly et al., 2021; Montena et al., 2021) show promise, including telehealth programs (e.g., through video, phone, and text modalities) linking veterans to primary care, mental health care, specialty care, and addiction treatment (Brunet et al., 2020).

Video telehealth visits, specifically through the VA Video Connect app (hereafter “video visits”), offer a potential avenue for veterans experiencing homelessness with SUD to access clinical services (Jacobs et al., 2019; McInnes & Cutrona, 2018; Miller et al., 2016). A recent systematic review found that mobile health interventions for the prevention of alcohol and other substance misuse are feasible and effective (Kazemi et al., 2017). A timely study found that many veterans with SUD who received Apple iPad tablets (hereafter “tablets”) for VA telehealth prefer video visits to in-person visits (Slightam et al., 2020). However, gaps remain, as veterans with a SUD generally receive fewer video visits than those with other types of mental health disorders (Grubbs et al., 2015). As well, clinician attitudes regarding telehealth quality and ease of use have been shown to influence the modality offered to patients, with two-thirds of mental health clinicians preferring video visits over phone, compared to primary care and medical/surgical specialty clinicians who preferred phone visits or had no preference, citing video visit challenges (Connolly et al., 2022).

VA video visits have seen exponential growth, yet adoption has remained uneven — with vulnerable veterans substantially less likely to use it (Ferguson et al., 2021). To address this, in 2016, VA Office of Rural Health collaborated with VA Office of Connected Care to expand video visits through the VA Video Connect app (Heyworth et al., 2017; Zulman et al., 2019). These program offices initiated nationwide tablet distribution to overcome barriers experienced by low-income and/or rural veterans to accessing in-person or telehealth care, at VA or in the community. Barriers include long travel times, few transportation options, and limited or unaffordable broadband connectivity (Heyworth et al., 2020; Slightam et al., 2020; Zulman et al., 2019). VA providers referred eligible veterans through a standardized consult in the electronic health record. Between April 2015 and October 2022, more than 35,000 veterans with a history of housing instability received tablets (VA-QUERI, 2022). Tablets come pre-loaded with the VA Video Connect app (for participation in video visits), wireless service plan, and Wi-Fi/4G mobile connectivity (Zulman et al., 2016). With few limits, veterans can use tablets to access useful resources at VA and beyond, such as housing services, food pantries, clothing, and job search. In 2020, VA added its “White Glove” phone support, providing set-up and testing of these devices with veteran recipients (VHA Telehealth Services, 2022).

However, the authors’ recent research found that access to a mobile device is often insufficient for video visit adoption. Among 1470 homeless veteran tablet recipients, fewer than half (46%) completed a video visit within six months of tablet receipt (Garvin et al., 2021a). Having a SUD was associated with an additional 41% reduced likelihood of tablet use — an important finding given the 60% prevalence of SUD in veterans experiencing homelessness (Edwards et al., 2020). Neither tablet receipt nor subsequent equipment loss (< 4%) explained the low rates of video visits.

Hence, the purpose of this study was to design an intervention tailored to the unique needs of veterans experiencing homelessness with SUD that supports their increased and sustained use of video visits. Findings may contribute to initiatives that offer use of video telehealth devices as an alternative to in-person healthcare encounters in post-pandemic behavioral health care.

## Methods

Telehealth interventions can initially face low patient engagement due to user attributes (including patient perception) and the intervention content, design, and context. The Unified Theory of Acceptance and Use of Technology (UTAUT), an established model in health services research, addresses these factors (Venkatesh et al., 2003). Hence, this qualitative study applied UTAUT to categorize veterans’ and experts’ views and experiences using a tablet for video visits, as well as barriers and facilitators to adoption and use. The four UTAUT determinants of a technology’s use are its perceived: performance expectancy (i.e., usefulness), effort expectancy (i.e., ease/convenience), social influence (e.g., relationships with family or doctor), and positive or negative facilitating conditions (i.e., staffing or technology resources). These determinants informed the study’s three phases: (1) veteran interviews to identify barriers and facilitators to video visit use and to propose intervention candidates; (2) an expert panel of providers to advise on patient telehealth needs and potential interventions, and veteran focus group to determine an intervention candidate; and (3) intervention mapping to design a prototype.

The local institutional review board approved study design and procedures. Veterans provided verbal informed consent for participation and audio recording. VA clinical researchers provided verbal informed consent to participate. The study was conducted from January to December 2021.

### Phase 1: Veteran Interviews

As previously reported, (Garvin et al., 2021b) purposive sampling was used on a national sample of 210 veteran tablet recipients. A study mailing to veterans was followed by phone recruitment. To focus on video visit concerns related to homelessness and SUD, eligible veterans had in the past year experienced homelessness with SUD only, i.e., veterans who additionally had depression, post-traumatic stress disorder, and/or serious mental illness were excluded. The study team conducted 28 1-h, semi-structured qualitative interviews by phone with veterans about their use (or non-use) of tablets for video visits. Interviews were audio-recorded with veteran verbal consent and transcribed verbatim. Participants were reimbursed with a $25 gift card for participation.

For analysis, transcripts were transferred into NVivo, a qualitative data analysis software package (QSR International, NVivo 9). A codebook was developed, with a priori codes consistent with the four UTAUT constructs, adding codes for any themes that emerged from the transcripts. Interview results were synthesized using thematic analysis to identify patterns and relationships (Braun & Clarke, 2006, 2014).

### Phase 2: Provider Expert Panel and Veteran Focus Group

#### Provider Expert Panel

Based on findings from veteran interviews, an expert panel was convened with four leaders from the VA Office of Mental Health and Suicide Prevention and the VA Center of Excellence in Substance Addiction Treatment and Education, all with clinical and research experience with homeless veterans with SUD. We sought to obtain their feedback on veteran interview findings, to add their insights on barriers to virtual care for veterans with a SUD, and to solicit ideas for other potential interventions. The meeting was recorded and transcribed. Their feedback and suggestions for additional interventions were then integrated with findings from veterans to develop candidate interventions.

#### Veteran Focus Group

We conducted a 1-h virtual focus group composed of five members of the Veteran Engagement in Research Group (VERG) at the Center for Healthcare Organization and Implementation Research at the VA Medical Centers in Boston and Bedford, Massachusetts. (VERG) These veteran consultants prioritized the intervention candidates proposed by veterans in phase 1 and provider experts in phase 2. To be eligible, all veteran members were required to have previous experience of either homelessness or VA behavioral health treatment, such as for a SUD, or both.

The goal of the focus group was to assess candidate interventions developed from phases 1 and 2 findings (Bowen et al., 2009; Pyke-Grimm et al., 2011; Vaughn et al., 2019). The protocol included an introductory statement, 6–8 open-ended questions on the perceived impact of the interventions on the four UTAUT factors, follow-up questions (or probes), and closing statement. Participants were encouraged to speak from their own experience and to suggest improvements to the interventions. With participant verbal consent, the focus group was audio-recorded and transcribed verbatim for analysis. Participants were reimbursed with a $25 gift card for their participation. Phase 2 qualitative analysis was similar to phase 1: transcripts were transferred into NVivo, and a codebook with a priori codes based on UTAUT constructs was used to categorize feedback. Additional codes were added for any themes that emerged from the transcripts.

### Phase 3: Intervention Mapping

Based on phase 2 findings, intervention mapping (IM) was used to design a promising intervention. IM is a protocol for planning and developing theory- and evidence-based health promotion interventions (Rollnick & Miller, 1995). In preparation, relevant VA Program Offices were engaged to advise on intervention goals. Six consecutive steps of IM process were followed to (1) create a needs assessment; (2) specify intervention performance objectives; (3) design the intervention based on evidence-based methods; (4) develop the intervention based on practical application; (5) develop a prototype implementation plan; and (6) prepare a performance assessment with specific success measures.

## Results

### Phase 1: Veteran Interview Results

Interviews with veterans experiencing homelessness with SUD revealed that the cooccurence of their health disorders, combined with low digital literacy, introduced unique barriers to video visits and other care (Garvin et al., 2021b). Veterans reported co-occurence of health- and military service–related conditions, including physical limitations (e.g., poor eyesight or hearing, hand tremors, disabilities) that hinder device utilization; and cognitive challenges (e.g., attention deficit disorder) and behavioral challenges (e.g., substance use) that diminish concentration and memory. For example, one veteran with a disability said his tablet password had been pre-set so that he would not need to alternately type upper and lower case letters: “They gave me the [tablet] password but I forget. It was lower case only ‘cause I have a hard time going back and forth on the iPad” [Participant #1]. Another veteran added “I have pain all the time.” [Participant #2].

#### Ease/Convenience

Of the four UTAUT determinants, ease/convenience was the most frequently cited advantage of video visits. Many veterans faced chronic conditions that impeded travel and social connection. Travel was particularly challenging for rural veterans whose closest VA facility was more than an hour away by car or public transit. VA’s telehealth tablets offered timely, convenient and cost-effective access to care and services. However, perceived complexity of the VA Video Connect app and tablets posed adoption barriers for some. Said one veteran, “VA needs to make the iPad as easy to use as the telephone” [Participant #3]. Another remarked: “The links to the visits used to be confusing. Now, they’re better” [Participant #4]. Lack of internet connectivity or privacy for a video visit in their living area were other barriers. “Where I live, the walls are thin; sounds travel,” shared one veteran [Participant #5]. A VA training call to set up the tablet was considered a facilitator.

#### Usefulness

Usefulness of video visits, hailed by veterans as a lifeline, was also a primary advantage. Among veterans who were able to operate the tablets they received, veterans appreciated their tablets’ reliability, generous data plan and good internet connectivity, which surpassed that of other devices that veterans owned. One veteran remarked: “I am comfortable with the iPad and like to have a VVC [VA Video Connect] visit with my doctor because we can see each other. It is person-to-person. You’re not just on the phone” [Participant #6].

#### Social Influence

A number of veterans were situated in VA-supported residences. They often reported living alone without much interaction with family, friends or the community. Thus, a secondary benefit of the video visits was reduced isolation through connection with VA providers, “a feeling of being heard and understood.” [Participant #7].

#### Facilitating Conditions (Positive and Negative)

The greatest need for improvement voiced by veterans experiencing homelessness with SUD was the perceived lack of VA support for training and adoption. Said one veteran: “I’m like—I can’t figure out how to work it [tablet] as far as setting it up” [Participant #8]. Veterans called for improvements to address: confusion with the visual layout of apps on the tablet’s opening screen, lack of awareness of VA Help Desk phone support, and lack of tablet training for veterans and providers. For example, despite the VA Help Desk’s practice of three outreach phone calls to help veterans with device set-up, veterans experiencing homelessness with SUD reported not receiving these calls, leaving many to complete device set-up on their own or, frequently, not attempting set-up at all. Some reported receiving the Help Desk call but found it insufficient, given their lack of digital skills. One veteran recalled: “I think it [tablet] arrived in the mail and I think I was told to call a number. This lady kind of explained a brief overview of some of the apps that were on there and that was it” [Participant #9].

The most frequent solution suggested by veterans was digital skills training provided by VA-employed peer specialists. As one veteran explained: “When you’re dealin’ with people that are somewhat ignorant to technology…then they are lost… I think we need more peer support….[The peers] need to be out and about in the community…you’re gonna trust it if you’re hearing it from one of your peers” [Participant #10]. Peer specialists are veterans who are far along in their recovery from addiction, mental illness, or homelessness who are trained by VA to serve other veterans. Many studies demonstrate the effectiveness of peer specialists in increasing patient engagement with mobile health applications by providing technical support on devices and apps, and encouraging accountability for continued use (Mohr et al., 2011; Ray et al., 2017), particularly for mental health support (Fortuna et al., 2018). Peer specialists (hereafter “peers”) have facilitated patient engagement with healthcare services, reducing perceptions of mental health stigma by providing empathic support consistent with treatment compliance (Chinman et al., 2015; Ray et al., 2017).

Veterans proposed three intervention candidates: (1) peer-led digital training and support (Abadi et al., 2020; Barker et al., 2018; Blonigen et al., 2020a, b; Montena et al., 2021); (2) an automated tutoring text from the VA Annie Text Messaging app that allows VA clinicians to text protocols to patients and view individual responses (Yakovchenko et al., 2021); and (3) a mobile app to guide video visits log-on (Blonigen et al., 2020a; Connolly et al., 2021; Kuhn & McGee-Vincent, 2020). Veterans also suggested that providers familiar with behavioral treatment for SUD might have further intervention ideas.

### Phase 2: Provider Expert Panel and Veteran Focus Group

#### Provider Expert Panel Recommendations

Based on phase 1 findings, an expert panel was convened with four leaders from the VA Office of Mental Health and Suicide Prevention and the VA Center of Excellence in Substance Addiction Treatment and Education, all with clinical and research experience with homeless veterans with SUD. After considering the three intervention candidates suggested by veterans, expert panel leaders added two: (4) a motivational interviewing intervention based on intrinsic personal goals (Miller, 1983; Rollnick & Miller, 1995) and (5) a contingency management intervention based on extrinsic monetary rewards (Dallery et al., 2019; DePhilippis et al., 2018; Getty et al., 2019; McPherson et al., 2018; Rash & DePhilippis, 2019). The resulting five intervention candidates are described in Table 1.

**Table 1:**
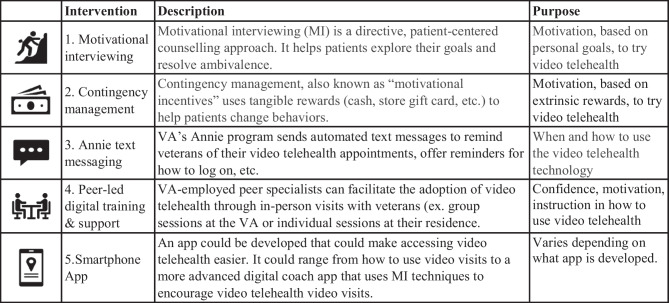
Intervention candidates to increase use of video telehealth among veterans experiencing homelessness with substance use disorder

#### Veteran Focus Group

In November 2021, a 1-h virtual focus group was conducted with five veteran members of the VA Bedford Veterans Employee Resource Group to prioritize the five intervention candidates. All veteran group members were male, aged 45–75, and the panel consisted of one non-White veteran. All had previously reported an experience of homelessness and/or VA behavioral health treatment. Veterans considered the merits of the interventions, both in the context of their own VA telehealth experience, and with regard to UTAUT constructs of usefulness, ease/convenience, social influence and facilitating conditions (as noted in brackets [], below). Veterans then voted for the intervention they deemed best and discussed their choices.

##### Smartphone App

One older veteran voted for the Smartphone App to guide veterans in how to log into a video visit. “It’s easiest. You just click.” [ease/convenience].

##### Automated Tutoring and Follow-up Text Messages

Another senior veteran voted for the Annie Text Messaging intervention, saying: “I have a bad memory. Those texts remind me about appointments,” [usefulness] though a third veteran countered: “Texts are annoying.” [inconvenient].

##### Contingency Management

The veterans overall did not believe that contingency management, which uses extrinsic motivation (e.g., financial payment), would be a good approach, suggesting that payment incentives “…wouldn’t work” [usefulness] and “are not sustainable” over time [negative facilitating condition: lack of organizational and/or financial support].

##### Motivational Interviewing

Instead, all of the veterans liked the intrinsic motivation inherent in motivational interviewing, saying that it aligned with VA’s patient-centered care approach (e.g., helping veterans to reach their personal health goals).

##### Peer-Led Digital Training and Support

A middle-aged veteran said: “I found peer support very helpful during a period of bereavement.” [usefulness]. Applying the concept of peer support to telehealth adoption, he continued: “I have a friend who is not computer savvy. He got some peer training on his tablet that helped him. But he needed multiple visits. You can’t expect some folks to pick it up on the first try.” [negative facilitating conditions: veteran lack of digital literacy and insufficient organizational support for training].

##### Combining Peer-Led Training with Motivational Interviewing

After a discussion of all intervention candidates, two of the five focus group members proposed a combination of peer-led digital training and support with motivational interviewing. The oldest veteran, who liked the combination, added: “Vets respond to someone like them. [social influence] Vets feel it’s worth their time if a peer specialist will talk them through it [digital training]…They need the coaching.” [facilitating condition: organizational support for training].

At the conclusion of the focus group, veteran members favored combining two intervention candidates: peer-led digital training and support, and motivational interviewing. Such a combination is consistent with the literature on VA peer specialist provision of digital training using a holistic approach like motivational interviewing (Fortuna et al., 2018; Mohr et al., 2011; Powell et al., 2012; Ray et al., 2017). Montena et al. (2021) offers further evidence for the promise of peers implementing innovation in behavioral health services. The authors suggest that employing peers in this way “further promotes… collaborative, patient-centered care coordinated across patient-specific presentations” (Montena et al., 2021).

### Phase 3: Intervention Mapping to Develop Prototype

#### IM Step 1: Needs Assessment

Based on phases 1 and 2 insights from veterans and provider experts, a needs assessment that prioritized the barriers and facilitators to use was created, including (1) patient background and healthcare experience; (2) physical, cognitive and motivational factors; and (3) patient experience expectations for video visits, informed by (4) UTAUT factors. The study team then composed and consulted an a priori list of types/modes of interventions relevant to video visit adoption, including: physician recommendation, live dialog, hotline or virtual agent (artificial intelligence-powered chatbot) to guide startup, educational materials (in print or online), phone hotline, text reminders, peer-to-peer coaching, or training sessions.

#### IM Step 2: Specification of Intervention Performance Objectives

To specify performance objectives, the study team first defined the two intervention components of peer-led digital training and support and motivational interviewing.

##### Peer-Led Digital Training and Support

Peers conduct in-person visits with veterans, either through group sessions at the VA, or individual sessions at the veteran’s residence. When possible, peers build on established relationships with veterans (e.g., arranging housing; supporting behavioral health treatment) to offer telehealth demonstration.

##### Motivational Interviewing

Peers use motivational interviewing (MI) to deliver digital training and support. MI was developed to help motivate patients to change hazardous substance use behavior, so it is well-suited to helping veterans experiencing homelessness with SUD (Miller, 1983; Miller & Rollnick, 2002; Rollnick & Miller, 1995). MI is a patient-centered, evidence-based method of communication to overcome ambivalence and promote behavior change. The approach is directive but nonjudgmental, empathic, individualized, and emphasizes the patient’s self-efficacy and autonomy (Walker et al., 2020). Among veterans, MI has been found effective in both individual and group settings (Santa Ana et al., 2021) and improves health behaviors (Britton et al., 2020). During the COVID-19 pandemic, MI has been successfully implemented via telephone to intervene with substance misuse (Walker et al., 2017).

**Fig. 1:**
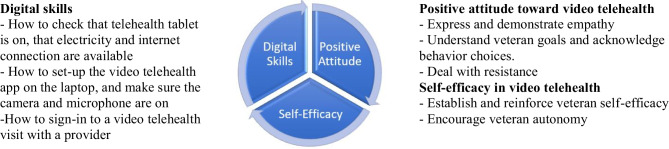
Model of three determinants to achieve performance objectives for a peer-led intervention using motivational interviewing

The study team then translated these into a model illustrating the digital skills to be taught (the “what”) and the positive attitude and self-efficacy (the “how”) of peer-led training and support intervention (see Fig. 1).

Next, the study team developed performance objectives for the processes of peer-led training and motivational interviewing, and for the outcomes: veterans’ digital skills, positive attitude and self-efficacy regarding video visits. These were formulated based on phases 1 and 2 veteran and provider expert findings regarding veterans’: (1) health and housing situations; (2) digital skills needed; (3) self-confidence in and attitudes toward telehealth use; and (4) social support for device use and to minimize isolation.

#### Training and Support Process Objectives

##### Digital Skills

Building digital skills involves a peer’s education of veterans on the basics of: checking device power and internet, set up of the VA Video Connect app on the device, and showing the veteran how to sign in for a visit. While seemingly straight forward, these tasks can be daunting for veterans experiencing homelessness with SUD due to a number of physical or behavioral challenges (e.g., lack of concentration, anxiousness; advanced age can hinder hand–eye coordination; substance use involving needles can reduce sense of touch in fingers).

##### Positive Attitude Toward Video Visits

To create a positive attitude toward video visits among veterans, (1) the peer expresses and shows empathy to build rapport and trust. (2) Next, the peer must elicit and understand veterans’ intrinsic goals (e.g., improving their health so they can build healthy relationships with their children). The peer helps the veteran to understand how behavior change (i.e., learning how to use telehealth) might serve these goals. If veterans make choices that take them away from their goals, the peer respectfully points out the gap between behaviors and goals. (3) Finally, the peer must deal with resistance to telehealth adoption. When veterans resist changing their behavior (e.g., willingness to log onto the tablet), the peer does not confront the veterans. In the course of the training session, the peer works with veterans to get them to examine different views and approaches, allowing veterans to choose their own approach. When resistance occurs, this is a sign for peers to try an alternative training approach.

##### Self-Efficacy Regarding Video Visits

To build veterans’ confidence in their digital skills and their desire to be autonomous in using video visits, peers can take two steps: (1) the peer can point to a veterans’ knowledge or success thus far regarding the tablet or visit sign-on as evidence to reinforce veteran self-efficacy. (2) The peer can develop a veteran’s autonomy by communicating that the authentic power to learn to use telehealth lies with the veteran, not the peer. Ultimately, veterans are responsible for their own behavior. Peers can guide and affirm as veterans develop a list of action steps to take to change their behavior (e.g., plan for their next video visit.)

#### Training and Support Outcome Objectives

Three outcomes that demonstrate the success of peer-led training and support include: Veterans’ digital skills (detailed below), their positive attitude, and their self-efficacy regarding video visits, all contributing to sustained use of video visits over time. Veterans are able to locate an electrical source and recharge the device on a regular basis. Veterans are able to locate and enter their device passwords. Veterans are able to independently set-up and maintain a working mobile device to the extent that the average consumer could (e.g., regularly updating versions of the VA Video Connect app or the device’s operating system). Veterans are able to sign-in and hold video visits with their healthcare teams.

#### IM Step 3: Intervention Design

In support of intervention design, the study team selected evidence-based methods from MI. These focused on peer training and support techniques that address veteran needs, context and personal goals to enable veterans experiencing homelessness with SUD in telehealth adoption and use. See Table 2, adapted from Bartholomew et al. 2006 and De Lepeleere et al. 2018 (Bartholomew et al., 2006; De Lepeleere et al., 2018).

**Table 2:** Evidence-based methods from motivational interviewing and peer training and support approaches to address determinants of video telehealth use by veterans experiencing homelessness with SUD

**Motivational interviewing methods**	**Peer training and support approaches**		
	**Digital knowledge/skills**	**Attitude**	**Self-efficacy**
Consciousness raising	Explain and demonstrate how to set up and log in for telehealth use by veterans	Express positive feelings about benefits of telehealth use, i.e., usefulness, ease and convenience, maintenance of social connection and image	Explain how the veterans’ own health and life goals can be achieved through use of telehealth
Imagery	Offering familiar physical or verbal images as analogies to a less familiar process		
Active learning		Encourage learning from social interaction- and activity-based experience	Encourage learning from goal-driven and activity-based experience
Active listening	Build trust and rapport through active listening, followed by summarizing and relaying back what the veteran said		
Guided practice	Prompt veterans to rehearse and repeat the behavior various times, discuss the experience, and provide feedback		
Verbal persuasion		Express affinity and encouragement for the veteran in their trial efforts to adopt telehealth	Express confidence in the veteran to be able to learn and maintain use of telehealth
Rationale			Propose one or more meaningful reasons and a logical conclusion
Modeling	Provide a role model for telehealth understanding, appreciation and use that reinforces the desired action		

#### IM Step 4: Intervention Development

**Fig. 2:**
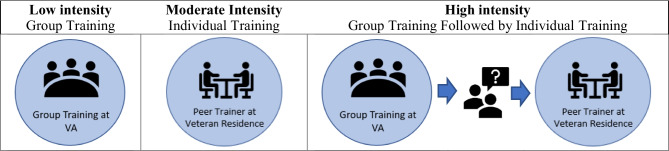
Stepped peer-led intervention to increase video telehealth tablet use by veterans experiencing homelessness with SUD

The study team developed a stepped peer-led training intervention that reflected the four UTAUT determinants of use (usefulness, ease/convenience, social influence, and facilitating conditions) and could be adapted for group and individualized training (Santa Ana et al., 2021). “Stepped care” refers to increasing intensity of peer-led training and support (Edelman et al., 2017, 2019), and such a program is designed to be flexible and provide increasing support based on the needs of individual veterans (see Fig. 2). If a veteran was reasonably able to travel to a VA facility (i.e., no mobility or travel distance challenges), a low intensity group training at a local VA facility would be offered. If the veteran faced mobility or travel challenges, or was disinterested/anxious about group trainings, then moderate intensity individualized training would be offered, with the peer traveling to the veteran’s residence or nearby location. Finally, if veterans who received group training still had difficulty using video visits, they would receive a follow-on individualized visit from a peer for high intensity stepped training.

#### IM Step 5: Implementation Plan

##### Training the Peer

Prior to holding group or individual training sessions with veterans, a member of the study team educates the peer specialist on digital knowledge and skills imparted in the training, covers the theory and practices of motivational interviewing, and reviews the motivational interviewing treatment integrity (MITI) protocol by which they may be assessed (see IM Step 6).

##### Veteran Group Training and Support

The peer’s role is to engage up to 10 veteran group members in an empathic and collaborative 1-h training session, focusing on veterans’ intrinsic goals, digital skill needs, and behavior change targets, evoking change talk, resolve ambivalence, and depending on readiness for change, initiate change planning.

For resources, each VA facility offering training arranges for a conference room which is wheelchair accessible and convenient to the building entrance, public transit and parking, and to rest rooms. The room offers amply-spaced seating and audio visual equipment. A training guide and materials for the peer (PowerPoint slides and handouts) covers digital knowledge/skills steps and motivational interviewing methods to elicit positive attitude and self-efficacy in veterans. The guide and materials are specifically designed for veteran participants who are currently/have experienced homelessness with SUD, and any co-existing behavioral health conditions. Group members are introduced to the peer-led classroom ‘culture’ including MI-consistent group norms for maintaining MI spirit (e.g., avoid giving unsolicited advice). Unlike individual training sessions, peers are mindful to promote constructive group behaviors including group cohesion, reinforcement of positivity, universality, and identification (Santa Ana et al., 2021).

##### Individual Veteran Training and Support

The peer’s role and training materials used with individuals is similar to that in group training (above). However, instead of a classroom, the peer requires a car to drive to the veteran’s residence or other location (e.g., local library, VA cafeteria) to provide digital support. Feasibility barriers the peer may encounter could include that the veteran’s apartment intercom does not work, or the veteran fails to keep the appointment. Feasibility barriers the veteran may face could include lack of electricity, internet, or privacy for a video visit.

#### IM Step 6: Performance Assessment

##### Training and Support Process Assessment

For training process assessment, the Motivational Interviewing Treatment Integrity (MITI) protocol, version 3.1.1 (Moyers et al., 2010) is a validated instrument that can assess a peer’s MI adherence and competence in individual and group settings (D'Amico et al., 2012; Santa Ana et al., 2021). The MITI measures two elements: global dimensions (empathy, direction, and MI accord, assessed on a Likert-scale (1 = low adherence; 5 = high adherence), and MI behavioral frequency counts (giving information, MI adherence, MI non-adherence, open/closed questions, simple/complex reflections). Summary scores gauge a peer’s MI capabilities in four function areas: percent MI adherence, open questions, complex reflections; and ratio of reflections to questions. Peers require MITI training as part of their initial training.

##### Training and Support Outcome Assessment

Ultimately, the effectiveness of the intervention is demonstrated by veterans’ ability to: find and secure electrical and internet access; set up the VA Video Connect app; and log in to a video visit. The intervention program (its supervisor and peer trainer) is assessed based on veterans’ successful completion of the training sessions and veterans’ sustained video visit use (potentially tracked over a 6-month period). As an efficiency goal, successful completion of the training is achieved, when pedagogically appropriate, through the less resource-intensive group training option.

## Discussion

Veterans experiencing homelessness with SUD often have co-occurring physical, mental and behavioral health conditions that can complicate diagnosis, treatment, and disease progression. Hence, ensuring their access to video telehealth, a modality they prefer, is vital. This study demonstrates how expert and veteran views contribute to the design of a tailored intervention to support veterans experiencing homelessness with SUD in their increased and sustained use of video visits. Veteran interviews revealed extensive healthcare needs, and barriers and facilitators to video visits, with veterans proposing three intervention concepts. A provider expert panel then recommended two more concepts, and a veteran focus group selected two among the five concepts. These were combined through intervention mapping to develop a prototype for future piloting. Thus, the health needs of these veterans were considered at each design stage.

This intervention is tailored to the needs of veterans experiencing homelessness with SUD, offering five unique advantages to support their increased and sustained use of video visits: (1) motivational interviewing, shown effective in SUD treatment, is combined with (2) in-person training and support by peers with experience of homelessness and/or SUD, engendering trust that permits peers to work closely to build veterans’ self-confidence. (3) This allows peers to strengthen veterans’ digital skills and positive attitude toward video visits that may sustain their use for the long term, and encourage use of other virtual health applications (e.g., VA’s My Health*e*Vet patient portal, Annie Text Messaging, and Mobile Apps). (4) This intervention offers flexible “stepped care” that provides increasing support based on the needs of individual veterans. (5) Finally, it can flexibly accommodate individual and group training formats, and be delivered at either a VA facility or the veteran’s residence.

Several study limitations should be noted. Our findings focused on veterans experiencing homelessness with SUD within the VA system, so may not be generalizable to other patient populations or health systems. While a provider expert panel was consulted, the perceptions of healthcare staff, often seen as telehealth “gatekeepers,” were not collected in this study (Cowan et al., 2019; Whitten & Mackert, 2005). Our investigation focused on video visits, so findings may not be applicable to other modalities (e.g., phone, text). Future studies should pilot the peer-led digital training and support intervention, assessing usefulness, ease of use and acceptability by patients, and usefulness and feasibility among providers and the healthcare system.

Importantly, this intervention supports VA’s broader commitment to health access and health equity through expansion of digital literacy. These research findings may have implications and benefits for other veteran or patient groups who face access barriers due to mental and behavioral health conditions, age-related ailments or other conditions (Wray et al., 2022). The collaboration represented by this study is one step in the greater consideration of social determinants of health and how to support the use of telehealth tablets to help cross the digital divide in health, and to reduce health disparities that are so prevalent among veterans experiencing homelessness with substance use disorder.

## Data Availability

Patient training materials are available from the authors upon written request.
